# Did the COVID-19 Pandemic Affect the Stress Levels among the Mothers of Premature Infants? A Narrative Review of the Present State of Knowledge, Prevention Strategies, and Future Directions

**DOI:** 10.3390/ijerph21081095

**Published:** 2024-08-19

**Authors:** Agata Trześniowska, Emilia Wagner, Alicja Ściseł, Kinga Szymańska, Karol Szyprowski, Żaneta Kimber-Trojnar

**Affiliations:** Chair and Department of Obstetrics and Perinatology, Medical University of Lublin, 20-090 Lublin, Poland; trzesniowska.agata1@gmail.com (A.T.); alicja.weronika.scisel@gmail.com (A.Ś.); kingasz21@gmail.com (K.S.); ka.szyprowski@gmail.com (K.S.)

**Keywords:** COVID-19, stress, mothers, NICU, premature

## Abstract

Understanding COVID-19’s effects on susceptible populations remains essential for clinical implementations. Our review aimed to examine whether the pandemic significantly impacted the stress levels in the mothers of premature infants in NICUs. The review of the literature from Google Scholar and PubMed resulted in identifying specific stressors such as the disruption of healthcare systems, limited access to neonatal care, uncertainty due to frequent changes in restrictions, the risk of COVID-19 infection, social isolation, and financial stress. While some quantitative studies concerning this topic did not show a significant increase in the perception of stress in this population compared to the pre-pandemic group, various research has indicated that the COVID-19 pandemic may result in enduring impacts on the emotional and neurological development of children. This article demonstrates a correlation between the repercussions of the COVID-19 pandemic and an elevated incidence of depressive symptoms among the mothers of premature infants. Further studies are needed to assess the long-term impact of pandemic-induced stress.

## 1. Introduction

### 1.1. Overview of COVID-19

COVID-19, caused by SARS-CoV-2, first emerged in Wuhan, China, in December 2019. By March 2020, the World Health Organization (WHO) declared it a pandemic [1]. The virus is primarily transmitted through respiratory droplets; the incubation period can range from 2 to 14 days, complicating early diagnosis and treatment [2]. The disease was found to be highly contagious, with significant community and familial transmission, which could occur even before symptom onset. Common symptoms of COVID-19 include fever, cough, shortness of breath, and muscle ache [3]. The virus primarily targets the lower respiratory tract, and severe cases can lead to ARDS and multi-organ failure, especially in older adults and those with underlying health conditions [2].

The COVID-19 pandemic led to increased social isolation and loneliness, particularly among the elderly and youth, resulting in heightened anxiety, depression, and neurocognitive decline. Mental health disorders such as anxiety, depression, and post-traumatic stress symptoms surged due to fear, economic instability, and prolonged stress. Substance use, including alcohol and cannabis, rose as a coping mechanism, especially among middle-aged adults. Vulnerable groups like essential workers, healthcare providers, and individuals from lower socioeconomic backgrounds face higher mental health risks [4].

### 1.2. Preterm Birth and Challenges

Preterm birth, occurring before the 37th week of pregnancy, presents significant challenges for mothers and is a leading cause of perinatal mortality and long-term morbidity in newborns [5]. The risk factors for unplanned preterm births include racial disparities, periodontal disease, low maternal body mass index (BMI), and previous preterm births [5]. The most reliable indicators of preterm birth are a short cervical length and a high cervical–vaginal fetal fibronectin concentration [5]. Poor maternal mental health during pregnancy is associated with a higher risk of preterm birth (PT) and low birth weight (LBW) in offspring. This suggests that the mental health of mothers is a significant factor in the challenges related to preterm births [6]. Women who are at risk for preterm delivery should be identified early and offered access to effective treatments [5,7]. The conditions of preterm delivery often prevent mothers from seeing their baby immediately after birth, which can hinder the initial bonding process [8]. The initial shock of an unexpected early birth and the subsequent stay in the neonatal intensive care unit (NICU) can be traumatic, leading to prolonged feelings of grief and loss [9]. Mothers may struggle to recognize their baby as the one they imagined during pregnancy, leading to difficulties in forming a bond. The presence of medical equipment, such as incubators, tubes, and masks, can create a physical barrier between the mother and infant. This can make mothers feel disconnected and insecure about interacting with their baby, further complicating the bonding process [8]. Parental mental health is a crucial factor affecting child development. The evidence suggests that mothers of children born preterm may require support not only in the initial months after birth but also later in the child’s development [9].

### 1.3. Research Focus: COVID-19 Impact on Stress Levels of Mothers of Preterm Infants

The impact of the COVID-19 pandemic on the stress levels in mothers of preterm infants has been a significant concern [10]. The pandemic led to restrictions that affected the parental presence in neonatal intensive care units (NICU), which in turn increased stress and mental health problems among parents and families [11]. Mothers experienced accumulative stress due to a combination of concerns for their infant’s health and COVID-19-related stressors. This included the dissonance between their expectations of birth and the reality they faced, fear of infecting their infant, loneliness, and stress from the restrictions disrupting their daily routines [12]. The separation from their infant and the lack of physical closeness were significant stressors [13]. Maternal stress during the pandemic negatively affected women’s mental health, exacerbating existing pathologies or increasing cases of anxiety, depression, or stress [10].

### 1.4. Objective

The purpose of our article is to examine how the COVID-19 pandemic affected stress levels among mothers of premature infants in NICUs.

## 2. Materials and Methods

Sources (both quantitative- and qualitative-phenomenological studies) were gathered by using the Google Scholar and PubMed search engines and typing in the keywords. The time criterion for most papers was set to “since 2020”. Papers older than eight years were used only by exception due to their overarching nature.

## 3. The Current State of Knowledge

### 3.1. Understanding Stress in Mothers of Preterm Infants

#### 3.1.1. Factors Contributing to Maternal Stress in Preterm Births

- Emotions:
  The sudden onset of labor and the birth of an extremely preterm infant lead to an emotional crisis, characterized by feelings of dread, anxiety, and fear of losing the newborn. Mothers experience a period of emotional shock and a sense of emptiness, as they are not prepared for the premature separation from their child [14]. The physical environment of the NICU has been associated with health issues, personal restrictions, and lower levels of attachment, especially among mothers [15]. Mothers of preterm infants experience a range of emotional distress, including feelings of sadness, fear, anger, the loss of self-esteem, and a sense of failure and guilt for their child’s pain. The stress and postnatal depression experienced by these mothers can have enduring consequences on maternal–infant parenting behaviors [16]. Mothers reported significantly higher anxiety levels and felt more personally restricted than fathers, indicating a higher emotional burden [15]. Younger mothers and first-time mothers reported higher stress scores, suggesting that less experience can provoke more stress [16]. The perception of stress in this group is also influenced by ethnicity and the related differences in social support (research shows that minority women are at risk of experiencing more stress and developing symptoms of depression) [17].
- Medical concerns and NICU experiences:
  The sudden concern for the health and developmental problems of a prematurely born child was a source of considerable stress for parents [15]. The NICU environment is inherently stressful, with preterm infants undergoing numerous acute procedures and chronic events, particularly in the first few days of admission. The presence of healthcare professionals, loss of the parenting role to the medical team, and the complexity of the NICU environment contribute to maternal stress [14].
- Bonding challenges and role confusion:
  The experience of a preterm birth can disrupt or delay the bonding process between parent and infant. Bonding is influenced by factors such as the physical proximity between parent and infant, the emotional state of the mother, and the infant’s ability to communicate [15]. Maternal role alteration, especially being separated from their infants and not being able to feed them, was identified as the most stressful experience. This reflects the stressful feelings of mothers who are deprived of the most pleasurable aspects of infant intimacy and care. Practices that promote closeness and earlier physical contact between mothers and infants have many physiological and emotional benefits and can help buffer against the stress of separation, aiding in the recovery of the infant and supporting the parent–infant bond [14].

#### 3.1.2. The Importance of Maternal Mental Health in Neonatal Care Outcomes

The adverse impact of negatively affected perinatal maternal stress on infant development during the pandemic is a serious concern. Research has demonstrated that stress affects infant development, indicating that infants with better regulatory abilities are associated with reduced parenting stress and enhanced mother–infant bonding [18]. Additionally, maternal stress impacts fetal central and autonomic nervous system function, hypothalamic–pituitary–adrenocortical axis activity, and fetal HPA development, which can influence the early structures of the developing limbic system, such as the amygdala and hippocampus [18].

Parental presence in the NICU is associated with benefits for preterm infants, including more stable physiological responses, improved oral feeding, and a reduced length of stay. The more stressed parents are, the less able they are to support their children, which can have a significant impact on the premature infant [15].

### 3.2. COVID-19 Pandemic and Maternal Stress in Preterm Births

#### 3.2.1. The Disruption of Healthcare Systems and Access to Neonatal Care

The quality of prenatal, natal, and postnatal care has been found to be negatively affected by the COVID-19 pandemic [19]. Significant shifts in the maternity care setting have impacted women’s capacity to make independent decisions regarding their birth and have changed their sense of security [20]. In an effort to protect both mothers and infants, the prenatal and postnatal care policies in the hospitals required careful revision. This included the cancellation of non-essential consultations, check-ups, and prenatal classes, as well as limiting the attendance of the women’s partners during prenatal consultations, and prohibiting family members and visitors from postpartum units, leading to social isolation for the mothers [21]. The number of obstetric-related cases presented to emergency departments has declined, and there have been limitations in the availability of health care tests and treatments [18].

Widespread changes in hospital entry screening policies during the COVID-19 pandemic also affected NICU entry [22]. Restricting parental presence further exacerbated the emotional turmoil experienced by families in the NICU, who were already susceptible to psychological distress due to the traumatic experience of being separated from their infant and the anxiety over their medical condition. COVID-19-related policies that restricted aspects of parental presence in the NICU may have undermined the concept of parents as an active party involved in care, contrary to the principles of Family Centered Care (FCC) [18]. Essential family-centered developmental care interventions and practices were disrupted, leading to a decrease in the active participation of parents in their infant’s care [23]. The changes in the developmental care activities that were delivered not only affected parents but also clinical staff, who were not subject to visitation policy restrictions [24]. Healthcare professionals, who previously used to advocate for FCC before the pandemic, stopped involving parents in care, placing parents in a stressful situation [25].

The COVID-19 pandemic significantly affected staff organization, with implications for infant care. Staff redeployment and delays in non-urgent procedures, such as multidisciplinary therapies, occurred [22,26,27]. The redeployment resulted in a decrease in on-site clinical family support services, which are crucial for identifying and initially supporting parents who are experiencing psychosocial distress [23,24]. The developmental team, in addition to providing direct patient care, plays a vital role in teaching parents about essential daily techniques for both in-hospital and post-discharge care [22]. However, due to a lack of staff support and imposed restrictions on the time spent with their infants, parents often faced a tough choice between learning the necessary technical skills for proper infant care and bonding with their child [27].

Moreover, the use of personal protective equipment, virtual consultations, and online antenatal education disrupted interpersonal communication and limited the supportive contact between NICU staff and parents [18]. The heavy workload, increased levels of stress, and personal pandemic-related issues of medical staff visibly affected their availability and patience towards parents [12]. In situations where both the healthcare professionals and new parents were under substantial mental health strain, restrictions on interpersonal interaction could have potentially exacerbated the emotional fatigue experienced by expectant women, new mothers, and their families [18].

#### 3.2.2. Increased Anxiety and Uncertainty Due to Pandemic-Related Factors

During the COVID-19 pandemic, parents commonly experienced uncertainty, with a pressing need for information during this time [28]. The feelings of uncertainty due to the pandemic may have amplified the pre-existing emotional distress caused by the separation from the baby and lack of the partner support [29]. Mothers were forced to confront their expectations with the new and ever-changing reality. The ongoing uncertainty, not only regarding the infant’s fluctuating condition but also due to the rapidly evolving pandemic-related regulations, hindered the parents’ efforts to adjust, resulting in reported exhaustion and emotional fatigue. The prolonged hospitalization in the NICU, coupled with the intense stress stemming from the unpredictable and uncontrollable nature of the situation, along with the loss of crucial social support, may have rendered mothers of preterm infants more susceptible to psychological distress, depression, and post-traumatic stress [12].

Due to visitation restrictions, parents had a significant need for general information regarding their child, including their child’s status, progress in the process, treatments, and other aspects of their care. Additionally, they sought information about the pandemic and the reasons for restrictions [28]. Parents with heightened COVID-19-related concerns were found to experience greater stress and feel less connected to their children [18].

The fear of infection intensified the emotional burden and parental anxieties. Mothers expressed concerns about inadvertently transmitting the virus to their children, leading to apprehension about desiring contact with them, which provided an additional source of stress and anxiety [25,28]. There were also fears that not just they, but their partners or healthcare professionals, could serve as a risk of infection for their infants, escalating their sense of responsibility and anxiety [12].

Pregnancy during the COVID-19 pandemic has also been linked to an increased risk of stress and frustration. The prospect of delivering in hospitals that were teeming with patients who were infected with SARS-CoV-2 heightened the anxiety of mothers about the potential infection risks for themselves and their newborns, which could have had detrimental physical and psychological effects on both. The fear of virus exposure may have caused pregnant women to hesitate or delay seeking medical care, which could have led to less favorable outcomes [18].

Amid the pandemic, there was a prevalent fear among parents about returning home. They understood the significance of their children’s post-discharge follow-up and expressed concern that the pandemic may hinder this crucial process. Additionally, parents were aware of the potential health risks their infants may face at home and worried about not being able to seek timely medical advice due to transportation restrictions, indicating the presence of a high emotional strain, even after discharge [28].

#### 3.2.3. The Impact of Social Distancing Measures and Restricted Support Networks

The strict restrictions that were implemented in hospital perinatal environments, including the NICU, amplified the separation between parents and infants, as well as between parents themselves, which, coupled with the anxiety surrounding the SARSCoV-2 pandemic, has been linked to acute distress [27]. The decrease in family visitation and parental presence posed a risk to parent–infant bonding, the delivery of crucial parent-led care activities, and positive health outcomes for both parents and infants [24,26]. This resulted in heightened stress and mental health issues among parents and families, increasing the likelihood of postnatal depression and post-traumatic stress disorder [11]. Many mothers reported feelings of isolation, loneliness, and significant stress due to the COVID-19 restrictions and social distancing measures, which prevented them from having visitors, receiving emotional and physical support from their own mothers or partners, or interacting with other mothers in the NICU for mutual support [12].

For the majority of the parents, limitations on time and physical interaction with their infant due to preventive measures were a key factor that adversely affected their parenthood experience [13]. Mothers indicated that the restrictive policies on their NICU access hindered their ability to bond with their infant or to be involved in their infant’s care or NICU daily rounds [15,19,26,27]. During the “no visitors” policy, parents felt anxiety, helplessness, and symptoms of depression due to the lack of contact and closeness with their infants. The fear of potentially not being able to see their babies again contributed to significant distress [25]. Excluding parents from the caregiving, planning, and decision making related to their child is less likely to foster feelings of competency and a healthy parent–infant relationship [11].

Mothers tend to experience heightened stress when they are in the NICU without the presence of the father. Not being able to share NICU visits with their partner inhibits the mutual support between parents, which is a crucial resource for the emotional adaptation to such a distressing situation. The absence of a partner’s presence and support during labor, delivery, and the initial postpartum days has been associated with increased symptoms of anxiety and depression in perinatal women. The restrictions created conditions permitting only one parent to develop intimacy with the child, hindering emotional mutual support as a couple during difficult times. Parents found it challenging to form a family unit without the ability to build parental relationships through shared experiences with their infant, resulting in a sense of emotional loneliness [15,30]. Limited presence in the neonatal unit provided fathers with fewer opportunities to learn and gain confidence in caring for their child [28]. It served as a significant barrier to the early bonding between the infant and the father, leading to feelings of loneliness and isolation in mothers [18].

Amid the pandemic, visits by the parents to the NICU were curtailed, leading to less opportunities for nurturing experiences such as breastfeeding or kangaroo care, which are typically used to alleviate stress [13,27]. A decline in the frequency, quantity, and duration of the developmental care activities provided by both family members and clinical staff for preterm infants in NICU was observed [24]. This could potentially impact parents’ ability to manage pandemic-related stress, intensifying the stress of having a baby in the NICU and further undermining their confidence as primary caregivers [13]. Moreover, the inability to assist and support mothers in initiating breastfeeding within the first hour after birth, coupled with the refusal of breastmilk donations, adversely affected the mothers’ emotional state, self-esteem, self-assurance, and belief in their caregiving capabilities [18].

Parents also indicated that the requirement to wear face masks and protective gowns interfered with bonding with their infant and made interactions with staff feel impersonal [12,27]. Standard safety measures, like hand hygiene or regular disinfection, enforced on mothers who were suspected to have, or confirmed to have, COVID-19, might have placed additional psychological burdens on new mothers and potentially complicated the initial relationship between mother and infant [18].

The pandemic led to a significant reduction in social support due to the social isolation that was associated with the restrictive measures and repeated lockdowns [21]. Parents adopted measures such as restricting the entry of outsiders to protect their children at home from the fear of infection, simultaneously reducing the opportunities for receiving help [28]. The fears associated with the COVID-19 pandemic reduced parents’ openness and willingness to seek support, which in turn may have diminished parents’ resources for coping with stress and anxiety [13]. The inability to share their concerns face-to-face with other family members intensified the mothers’ feelings of carrying the responsibility alone [12]. Without adequate postpartum social support, many women were left feeling isolated and alone, a condition that potentially increases the risk of developing perinatal anxiety and mood disorders [18].

#### 3.2.4. Financial Stress and Socioeconomic Disparities Exacerbating Maternal Stress

The pandemic’s lockdown and shutdown measures resulted in people experiencing economic hardship, instability in or the loss of jobs, and uncertainty for their future economic status [18]. COVID-19’s impact led to a rise in unemployment among women, as well as to an increase in childcare responsibilities due to the closure of nurseries and schools. This situation can result in a reduction in family income, which has been notably linked to higher scores on the EDPS among postpartum mothers [21]. Other significant reported challenges included difficulties with transportation during the pandemic, professional obligations, and worries regarding managing medical costs post-discharge [27]. Families with more limited resources appeared to be particularly affected by the stress of the pandemic, leading to a decline in the quality of parenting and family functioning [24]. A correlation between women’s unemployment or low income and a higher risk of depression and/or anxiety has been found in some studies. Furthermore, the financial strain related to COVID-19 has been significantly linked to a higher probability of a clinically significant depression score [31].

### 3.3. Research Findings on COVID-19’s Impact on Maternal Stress

During the COVID-19 pandemic, attempts were made to study the parental stress levels in the NICU. Some research focused only on pandemic groups, while others compared the results with those collected before the pandemic. The characteristics of the studies concerning the topic of this review are presented in Table 1.

The main stress assessment tool used in those studies was PSS (Parental Stressor Scale): NICU. This scale was created by taking into account the stressors present in the NICU environment and it consists of three subscales: Infant Behaviour (PSS-IBA), Parental Role Alternation (PSS-PRA), and Sights and Sounds (PSS-SS) [32]. Parents determined the stress level caused by a specific indicator on a scale from 1 to 5 [26]. In the other study, the visual analogue scale (VAS) was used in the assessment of general stress levels [33]. Also, other types of questionnaires were applied to rate the COVID-19 pandemic’s influence on parental stress [13,33].

#### 3.3.1. Studies Assessing Stress Levels in Mothers of Preterm Infants during the Pandemic

In one study, 30% of the participants described the pandemic as extremely or very stressful [13]. Since this study included both the mothers and fathers of prematurely born infants, a comparison of indicators was made between both groups. Some of them were significantly higher in mothers, which leads to the conclusion that they experienced more stress than their partners. In another study, not only preterm births were considered, as only 43.5% of the included infants were born before 37 weeks of pregnancy (and were admitted to the NICU for this reason) [23]. The research method in this case was an online survey conducted in the U.S. between May 2020 and June 2021, which included women who had given birth within six months and whose infants had stayed in the NICU. In addition to measuring the amount of stress, the level of concern about the possibility of contagion and the impact of it on one’s health was also determined. The majority (61.9%) of participants experienced a significant level of stress. It was revealed that the distress felt due to COVID-19 was related to stress levels. These results allow us to conclude that the COVID-19 pandemic was perceived as an important source of stress.

#### 3.3.2. Studies Comparing Stress Levels before and during the Pandemic

A study that compared the stress levels of the mothers of infants who were born before 32 gestational age and were admitted to the NICU prior to and during the pandemic showed no significant differences in any of the components of the PSS:NICU index [26]. However, there were more individuals at risk of experiencing severe stress (57.1% versus 43.9%) in the pandemic group. The reported facility adopted protective measures like access to psychological care or the possibility for both parents to be present in the unit and participate in the infant’s care. Also, the use of masks was obligatory only in the presence of personnel, which had an impact on maintaining skin-to-skin contact.

Another comparative study used the Parenting Stress Index to assess the impact of various sources of parental stress [15]. Both mothers and fathers were enrolled in the study, while the results from both groups were analyzed separately. The two involved centers implemented comparable safety precautions during the pandemic: only one parent was allowed to stay with the infant at a certain time and the presence of other visitors was prohibited. When completing the survey, parents were instructed to focus on the most stressful period of hospitalization. Findings show that during the pandemic, bonding was associated with greater stress for mothers compared to the pre-pandemic group (6.3 ± 1.8 vs. 4.9 ± 1.8). Also, a feeling of personal restriction was higher during the pandemic.

#### 3.3.3. Scientific Approach to Identifying Specific Stressors Related to COVID-19 in This Population

Identifying specific stressors affecting parental psychological well-being is important in order to enable the appropriate direction of protective measures. One study showed a negative correlation between the PSS:NICU Sights and Sounds index and the Maternal Postnatal Attachment Scale only in the group surveyed during the pandemic [26]. Another study found the highest contribution of the Parental Role Alternation subscale to overall PSS:NICU stress levels [23]. The same was observed for parents of infants admitted to the NICU in the Netherlands [33]. It was also noticed that during the pandemic, establishing a relationship with the newborn was more difficult for mothers in comparison to earlier periods [15]. Therefore, these results suggest that one of the most important stressors was the difficulty of fulfilling the role of a parent and building a relationship with one’s child.

This could have been impacted by the pandemic strictures. In one of the above-mentioned studies examining the effects of specific COVID-19 restrictions on parents’ psychological well-being, the greatest level of stress was related to the reduction in the amount of time that parents could spend with the infant and the inability to simultaneously care for the neonate with a partner (it should be noted, however, that the study included not only parents of children born prematurely: median GA 28+2 weeks) [33]. This is another identified aspect associated with COVID-19 that affected the process of becoming a parent most negatively (reduced contact and time spent with neonate and concerns about infant wellness) [13]. Although, even before the pandemic, many research findings had already associated the highest levels of stress among mothers with Parental Role Alternation [16,34]; the necessity of isolation might have further exacerbated this condition.

#### 3.3.4. Addressing the Gaps in Knowledge and Understanding of Maternal Stress in Births during the Pandemic

The COVID-19 pandemic impacted birthing environments, much like other aspects of our lives. Policies related to the pandemic could have influenced birth satisfaction. Notably, separating mothers and infants during the COVID-19 pandemic was linked to reduced birth satisfaction due to the lack of support and presence of a birth partner [20].

A study by Breman et al. that was conducted on 388 women who gave birth during the COVID-19 pandemic in 2020 proved that the COVID-19 pandemic affected women’s birth experiences. The majority of the responses remarked that the changes in facility policies had a detrimental impact on their experiences, mainly due to visitor restrictions and impersonal interactions with hospital staff [35].

### 3.4. Implications for Clinical Practice and Support Services

#### 3.4.1. The Importance of Screening for Maternal Stress in Neonatal Care Settings

The multitude of stressors experienced by the mothers of premature infants in the NICU makes them a particularly vulnerable group for experiencing broadly defined stress. The earliest possible identification of mothers with high levels of stress is essential as it allows health professionals to intervene promptly and prevent the condition from worsening [36].

A study carried out in the US found that screening for the mental well-being of the parents of NICU patients at many centers was inadequate (18.24% of surveyed NICUs did not conduct any screening), and the method of screening was not standardized [37]. The use of a variety of questionnaires during screening was also highlighted in another study [38]. On the other hand, units that regularly conducted screening were more probable to refer patients for treatment (91% vs. 64%) [37].

Reliable screening, therefore, makes it possible to identify the patients at risk and implement appropriate measures to prevent the further development of the disease. In addition, incorporating screening into the routine activities carried out in the unit enhances its application [38]. Determining the hospitalization period during which screening should be carried out, and the instruments for this purpose, can contribute to simplifying the comparison of the results from different wards, for example, for research purposes [37,39]. On the other hand, by integrating the use of various screening tools at a particular center, we can obtain more data on the stressors affecting a given mother’s well-being. It enables tracing the interactions between them, which allows a better understanding of the sources of psychological problems and directing therapies to eliminate the stressors that prove to be the most challenging for a given patient [40]. Focusing only on screening for postpartum depression without including other conditions that affect maternal psychological well-being (such as stress) overlooks a large group of patients who may benefit from psychological counseling [39]. Broadening and standardizing the screening instruments in the NICU is therefore beneficial from a mental health and research perspective.

#### 3.4.2. Strategies for Addressing Maternal Stress during the Pandemic

- Telehealth support:
  Limiting close contact is one of the primary measures used to prevent the spread of infections. Pandemic restrictions forced attempts to practice medicine through telecommunication, which allows the screening and monitoring of mental well-being [31]. One such practice is telenursing, which is one of the strategies considered to be useful in providing support and can be applied in clinical practice to reduce maternal stress in NICUs [41]. During the pandemic, the information about the infant that was provided by medical staff via phone or video calls and the recordings of these calls were very important to parents and reassured them when contact with the infant was impossible [28]. This is consistent with the results of a study in which one group of parents received information about their infant’s health through video calls and phone calls but was not allowed in the NICU [42]. In this group, the separation from the infant caused considerably less stress (as measured by the Parental Stressor Scale—Hospitalised Infant) than in the case of parents with whom communication via video call was not applied, but who were allowed to stay on the ward for one hour a day. In another study conducted in the NICU of the Children’s Hospital of Philadelphia before the pandemic, parents were able to access images from the bedside camera at any time during the hospitalization of their infants [43]. Using this device markedly reduced the level of stress associated with separation. The effectiveness of these methods seems to support the validity of introducing telehealth support in clinical practice.
- Providing accurate information and reassurance about COVID-19 safety measures:
  Adequate communication with parents about the reasons for particular restrictions facilitates their accepting them and understanding their meaning [28,33]. Receiving information about the pandemic through the media or from medical personnel is mentioned as one of the preventive measures against the onset of symptoms of depression or anxiety in perinatal care [31]. On the other hand, inconsistency in communicating restrictions led to a sense of frustration among infants’ caregivers [30,33]. One study presented statements from parents who felt dissatisfied due to the incoherence of the information provided by staff regarding the pandemic [30]. Efficiently informing and encouraging parents to discuss their concerns could therefore be considered important for maintaining their well-being.
- Collaborative approaches involving healthcare professionals, psychologists, and social workers:
  Research indicates that parents of preterm children still experience significant mental health difficulties even two years after childbirth [44]. The additional strain caused by the COVID-19 pandemic is likely to worsen parents’ risk of acute stress and anxiety. During the challenging period of the COVID-19 pandemic, it was crucial to enhance support to reduce stress transmission [45]. However, healthcare professionals frequently overlook maternal stress in the neonatal intensive care unit (NICU) [36]. Doctors and nurses in NICUs, particularly during global health crises such as the COVID-19 pandemic, should remain vigilant about the emergence of postpartum depression (PPD) and anxiety in NICU mothers. Regular screening for PPD is essential and should be carried out [19].

It would be beneficial if NICUs considered temporarily increasing access to mental health support. This would assist parents in creating effective stress coping strategies [45].

In Estonian NICUs, known to provide family-centered care, the number of the restriction measures was not associated with mothers’ PPD symptoms. That may be related to the support factors in Estonian NICUs, such as single-family rooms, overnight stays by parents, and additional psychological and emotional support being provided to parents by healthcare staff [46].

Mothers in NICUs need focused care, guidance, and support [36]. The systemic review and meta-analysis conducted by Maleki et al. demonstrated different strategies for the provision of emotional support to the mothers of preterm infants that can be applied by nurses in NICUs. The strategies are family centered care, parent support and education programs, interpersonal psychotherapy, skin-to-skin care, newborn-individualized developmental care, spiritual care, and telenursing. NICU nurses primarily interact with the mothers of infants, so they are a group that can have a significant impact on their emotional support [41].

Another study presented postnatal recommendations for the promotion of maternal mental health during the COVID-19 pandemic. Some suggested guidelines include encouraging skin-to-skin contact, minimizing separation between the child and mother when feasible (based on clinical conditions), facilitating family communication through telecommunications, and re-evaluating psychological symptoms after discharge from hospital [47].

Certain research has investigated the effects of various interventions in reducing stress levels among the parents of premature infants. In a study conducted by Castel et al., the parents of premature infants were included in the psychological intervention program called TRT (Triadic parent–infant Relationship Therapy). This program involved 22 sessions and was based on attachment theory. The TRT approach has demonstrated effectiveness in reducing parenting stress [44]. The result of a Brazilian study proved that interventions of live music therapy in the NICU decrease anxiety, stress, and postnatal depression among the mothers of preterm infants [48]. Another study reveals the impact of relaxation techniques on stress. The mothers of preterm infants used a special, audio-assisted technique for 10 days. The final result of the study showed a significant decrease in stress scores among the mothers in the experimental group of the study [49].

The pandemic had a significant impact on certain mothers. While we cannot alter the past, we can offer further support to mothers who were negatively affected by the pandemic and ensure that in similar situations in the future, all families receive the necessary assistance [50].

A study by Venta et al. highlights that it is important to do more research on the psychological and biological effects of stress on the mothers of infants, because it might motivate making maternal stress screening a standard part of postpartum care [51].

### 3.5. Future Directions and Research Needs

#### 3.5.1. The Long-Term Effects of COVID-19 on Maternal Mental Health

Limiting parental presence leads to heightened emotions of sadness, fear, anger, and distress in parents, placing additional strain on their mental health. This situation elevates the risk of post-traumatic stress syndrome and postnatal depression [52].

Multiple studies investigating the effects of the COVID-19 pandemic on the development of postpartum depression in the mothers of premature infants present diverse outcomes. The findings from the studies are presented in Table 2. A screening tool used in these studies is the Edinburgh Postpartum Depression Scale (EPDS). The scale is effective at detecting emotional and cognitive postpartum depression (PPD) symptoms. Thirty points is the highest score than can be obtained. Scores above 13 require additional evaluation, and are cause for a referral to a psychiatrist or psychologist [19].

A study conducted by Ozdil M. aimed to screen for postpartum depression syndromes among the mothers of infants hospitalized in the NICU in two stages of the COVID-19 pandemic. In the total sample the percentage of women with EPDS scores > 13 was 11%. This study discovered that the severity of the COVID-19 pandemic could negatively impact the mental health of NICU mothers [19].

In a published Belgian study from 2021 that focused on the impact of the COVID-19 pandemic on PPD among the mothers of extreme and early preterm infants, they also used the EPDS scale, and the conclusion was similar to the previous study. It was proven that the risk of PPD (EPDS score ≥ 13) was much higher in the mothers of preterm infants who were assessed during the COVID-19 pandemic than in the control group of mothers assessed between 2017 and 2019 (26% versus 12%, *p* = 0.043) [21].

A similar conclusion was found in a Malaysian study which aimed to measure the association between breastfeeding attitudes and postpartum depression among mothers with premature infants during the COVID-19 pandemic. In this study, depression status scores revealed that 27% of mothers with preterm infants were at an elevated risk of depression. This is the highest result among the studies analyzed in this article [53].

An Estonian study by Itoshima et al. presented a different result. This comparative cohort study included the mothers of preterm infants before and during the COVID-19 restrictions. No significant difference was found in the median EPDS scores between groups (7.0 and 8.0, respectively). Supportive systems in Estonian NICUs may have protected mothers from PPD symptoms during the COVID-19 pandemic. Examples of the supportive systems are the family-centered care culture, single-family rooms, overnight stays by parents, and additional psychological and emotional support being provided to parents by healthcare staff [46].

Another study in which no significant differences were found in the depression scores between groups of mothers with preterm infants before and during the COVID-19 pandemic was a study by Manuela et al. [26].

Most of the reviewed studies have demonstrated that the COVID-19 pandemic influenced the risk of PPD among the mothers of premature infants. These findings highlight the importance of addressing mental health issues among postpartum women.

#### 3.5.2. The Long-Term Effects of COVID-19 on Child Development Outcomes

Different studies showed that parental stress can have lasting effects on parent–child interactions and the child’s development [15]. A recent study investigated the link between maternal perceived stress during the COVID-19 pandemic and infant regulatory issues. The findings revealed that mothers experiencing higher stress levels took longer to soothe their infants at night. Insufficient quality sleep can impede various aspects of infant development, such as memory, executive function, language skills, and overall cognitive growth. Additionally, it was found that maternal stress was significantly associated with increased infant crying and fussiness [54].

Research indicated that infrequent parental visits are linked to later behavioral issues in prematurely born children [15]. A study [22] noted that 85% of the NICUs that were restricting parental presence permitted only one parent to be present at the bedside. Regular parental involvement in the care of the premature infant fosters bonding and supports the infant’s development. Medical interventions are vital for premature neonates’ survival and well-being, but parental support and presence are equally crucial components of care [15].

Research indicated that during the COVID-19 pandemic, the mothers of preterm infants experienced reduced bonding [15]. In the study that focused on the impact of restrictions on parental presence, it was indicated that the number of NICUs allowing 24/7 parental presence decreased noticeably during the COVID-19 pandemic (85–53%) [22]. The restrictive visitation policies implemented due to pandemic were a significant obstacle to mother–child bonding [15]. Social bonds form the foundation for an infant’s emotional growth and influence their lifelong ability to manage stress, regulate arousal, and engage in cooperative interactions [55]. Recognizing the significance of early bonding in a child’s life, the preterm infants born during the pandemic could experience lasting effects on their emotional and neurological development [15]. The World Health Organization and UK guidance are advocating to keep newborns with their mothers as it is important for the development of the infant [52].

In a study conducted by Polloni et al., 73% of parents reported less physical contact with their infants due to the preventive measures [13]. Recent research suggests that the use of personal protective equipment such as face masks in neonatal care, along with parental separation, can affect parents’ ability to communicate with their infants. This impact may have implications for the development of speech and communication [52]. Maintaining close contact between infants and their parents is essential, as separation increases the vulnerability of these infants to the immediate risks of long-term complications [52].

Another consequence of parental stress related to the pandemic is the weakening of language development support in early childhood. The cause of this phenomenon is attributed to insensitive parenting practices [56].

Research findings suggest that maternal stress during the COVID-19 pandemic had significant implications for infant development, communication skills, and bonding. Prioritizing stress management and fostering parental presence are crucial to promote positive outcomes in infants.

## 4. Conclusions

The COVID-19 pandemic was a source of many stressors for the mothers of premature infants staying in the NICUs. Social distancing measures restricted parental participation in infant care (family visitation and parental presence), which affected parent–infant bonding. The disruption in the healthcare system related to limitations on the availability of medical tests and treatment may also have led to a deterioration of the mothers’ emotional state. A stress factor directly related to the pandemic was the persistent fear of infection. Mothers were affected by uncertainty due to the pandemic, which might have been exacerbated by financial stress and socioeconomic disparities.

The results of the quantitative studies are not compatible with each other regarding whether the mothers of premature infants in NICUs during the pandemic experienced significantly greater stress than the pre-pandemic group. The differences in the observed maternal well-being could be explained by the distinctiveness of the pandemic restrictions implemented by a given NICU (adopting a policy allowing bonding with the infant and adequate psychological support might have reduced the negative impact of the pandemic) and the applied methodology (the usage of different scales to assess stress levels or focusing on more stressful periods of hospitalization). However, high levels of stress were observed in this population and pandemic restrictions were mentioned by parents as a factor causing stress. Also, the tendency to experience severe stress by a greater number of mothers in comparison to the pre-pandemic group was present.

Thoroughly exploring the psychological and biological effects of stress on the mothers of preterm infants holds significant importance. By identifying and addressing maternal stress early, healthcare providers can better support both mothers and their infants. It is also crucial to emphasize a collaborative approach that engages both healthcare professionals and psychologists. Multiple studies propose diverse approaches to offering emotional assistance to the mothers of infants, such as family-centered care, live music therapy, psychological intervention programs, relaxation techniques, early screening, the usage of telecommunication, and providing parents with sufficient information about the ward rules.

## Figures and Tables

**Table 1:** The main characteristics of quantitative clinical studies concerning the stress level of parents of premature infants used in this article.

Study	The Location Where a Study Was Conducted	Date of the Study	Research Group
[13]	The Padua University Hospital NICU	From June 2020 to February 2021	44 parents (25 mothers) of 25 infants (median GA * 34 weeks, IQR 31–35)
[26]	The Geneva University Hospital (HUG) NICU	From January 2018 to February 2020	20 mothers of infants born before 32 GA
		From November 2020 to June 2021	14 mothers of infants born before 32 GA
	The NICU of the Level-IV perinatal center of the University Children’s Hospital, Medical University Vienna, Austria	During 2018 and 2019	40 parents of 30 infants born before 32 GA (23.3–31.4)
[15]	The NICUs of: - the Level-IV perinatal center of the University Children’s Hospital, Medical University Vienna, Austria; - the Level-IV University Children’s Hospital, University Medical Center Hamburg-Eppendorf, Germany.	From February to May 2021	81 parents of 46 infants born before 32 GA (22.9–31.7)

* GA—Gestational Age.

**Table 2:** Comparing research findings regarding the effects of the COVID-19 pandemic on postpartum depression in the mothers of premature infants.

Study	Depression Screening Tool	Research Group	Gestational Age (Weeks)	% of Women with EPDS Scores ≥ 13	Mean EPDS Score
[19]	EPDS	125 NICU mothers during a period of high number of COVID-19 cases	33–39 (median 36)	15%	7.5
		125 NICU mothers during a period of lower number of COVID-19 cases	34–38 (median 35)	7%	5.6
[21]	EPDS	34 mothers assessed during COVID-19	<32	26%	-
		Control group of 108 mothers assessed between 2017 and 2019	<32	12%	-
[46]	EPDS	55 mothers assessed during COVID-19	<35	22%	7.0
		Control group of 54 mothers assessed before COVID-19	<35	26%	8.0
[26]	EPDS	14 mothers assessed during COVID-19	<32	-	10.53
		22 mothers assessed between 2018 and 2020	<32	-	8.45
[53]	EPDS	248 mothers assessed during COVID-19	<37	27%	8.14

## Data Availability

Not applicable.
